# Rethinking Circular Hospital Design: A Systematic Review

**DOI:** 10.7759/cureus.110759

**Published:** 2026-06-12

**Authors:** Ramkrishna Mondal

**Affiliations:** 1 Hospital Administration, All India Institute of Medical Sciences, Patna, Patna, IND

**Keywords:** circular hospital design, design efficiency, doughnut design, hospital architect, morpho space, nursing unit design, panopticon, round hospital design

## Abstract

Why are most hospitals or buildings of a rectangular design only? Compared to the standard rectangular Nightingale nursing unit design, why has a circular/elliptical nursing unit or hospital design not been developed to date? This study reexamines the concept of circular hospital building design. It aims to understand the reasons behind the limited existence of circular hospitals/buildings and compare circular with shaped buildings and their pros and cons. The study proposes a design concept based on further investigation to make economical, structurally stable, and efficient circular hospitals with modern technologies to improve patient safety, quality, and proper space utilization. The literature search was conducted using the MeSH terms “Circular Hospital”, “Round Hospital”, “Hospital Design”, “Doughnut design”, “Circular Building”, “Round Building” from Web of Science, PubMed, Scopus, and Google Scholar databases, last accessed October 27, 2025. The study followed the Preferred Reporting Items for Systematic Reviews and Meta-Analyses 2020 guideline but was not registered. The inclusion criteria were strictly assigned as circular building or hospital design only. Only 19 records were screened out of 96,210 records analyzing the pros and cons of circular design hospitals. The results show that the concept of circular hospital design is old enough, with the most significant advancements in the 1960s, 1988s, and 2015. Advantages of circular hospitals include wider visibility; reduced staff travel time; cost-effectiveness; improved light, air, and ventilation; and more bed head space. Limited literature evidence was found, with major limitations including wastage of central core areas and construction complexity. The author proposes that the design concept of a central elevator and modern construction technology reduces the central core waste and construction complexity. Use of modern amenities such as pneumatic tubes, capsule transportation systems, and heating, ventilation, and air conditioning and a medical gas pipeline system can help avoid transportation delays, reduce travel time, and reduce human error, ultimately improving patient safety, quality, and operational efficiency in the long term.

## Introduction and background

Hospital design strives to increase efficiency; reduce construction, maintenance, and operating costs; and ensure patient happiness, comfort, and privacy. Nursing units, consisting of nursing workstations with patients, have a significant impact on the overall architectural value of hospitals. To accomplish these goals, healthcare structures or nursing units have been created in a variety of designs, such as rectangular, circular, Nightingale, and harness-type, among others. However, the nursing unit is the dominant influence on architectural form and character in hospitals. Staff walking distance, traffic patterns, usable area, and service diversity all have an impact on efficiency. The proportions of patient areas have an impact on design efficiency as well [1].

It is well known that today we are seeing different hospital buildings, mostly rectangular in design. Even in our day-to-day activities, we are mostly acquainted with rectangular buildings. According to Steadman, the question is why most buildings are rectangular only? [2]. In this article, the primary questions are in the context of hospital buildings and/or nursing units in terms of their operational efficiency, construction cost, operating cost, patient and employee comfort, privacy, and happiness. These include (1) Which is better? Circular, rectangular, or elliptical? and (2) Which type of nursing unit or hospital design is better based on size, bed number, etc.?

To answer the above research questions, the author first performed a literature review. The subsequent sections of the article describe the detailed methodology followed, the findings, and further situational analysis based on the literature review; no meta-analysis was performed.

## Review

Methodology

A literature review was conducted using the following MeSH terms or keywords: “Circular Hospital,” “Round Hospital,” “Hospital Design,” “Doughnut design,” “Circular Building,” and “Round Building.” The review followed the Preferred Reporting Items for Systematic Reviews and Meta-Analyses (PRISMA) 2020 guidelines [3]. Web of Science, PubMed, Scopus, and Google Scholar databases were searched without any filters applied for duration and type of article. Only English language and Boolean operators (AND & OR) were used. The databases were last accessed on October 27, 2025. Two volunteers with a postgraduate qualification in hospital administration were assigned for article screening. In case of any disagreement, the independent views of two volunteers, along with the author’s view, were considered to redress the dispute or any bias. The inclusion criteria were limited to circular hospital design and/or circular building design-related records and were screened according to the inclusion criteria of circular hospital design-related records only. The study duration started in 2022, after approval from the Institutional Ethical Committee in October 2025. The long delay was due to the collection of full-text records. A preliminary study was presented at the ISQua Brisbane October 2022 conference (submission identifier: ISQUA22-ABS-1298 with poster number: P217). The review was not registered with any registry.

Findings

**Figure 1:**
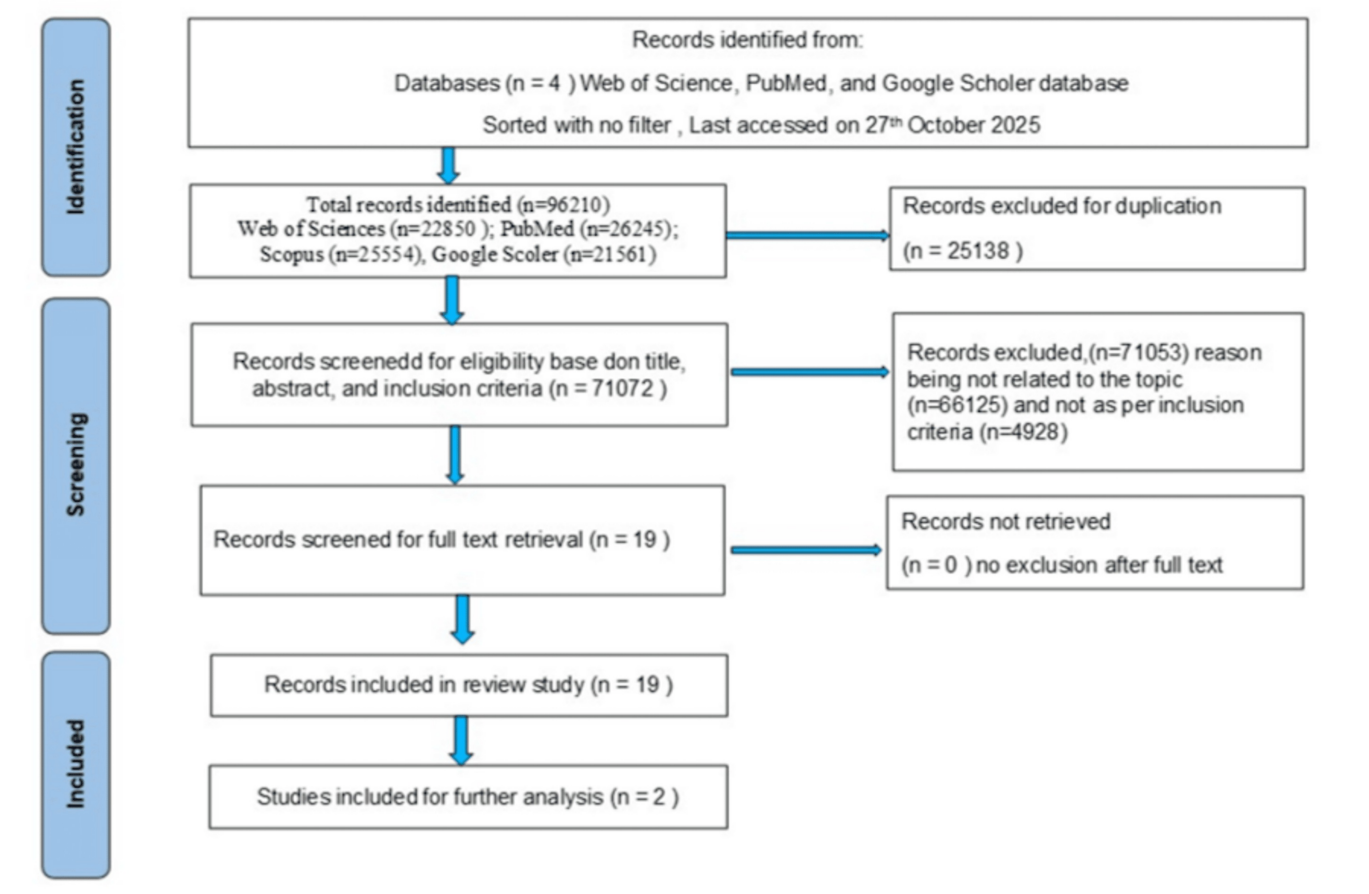
Systematic review methodology flow diagram as per the Preferred Reporting Items for Systematic Reviews and Meta-Analyses (PRISMA) guideline 2020. The figure was created using MS PowerPoint software.

According to the PRISMA 2020 guidelines, the systematic review search found a total of 96,210 records (Web of Science: 22,850, PubMed: 26,245, Scopus: 25,554, and Google Scholar: 21,561). After removal of 25,138 duplicate records, 71,072 records were screened. Detailed screening was done based on title, keywords, and abstracts following the inclusion criteria. Out of 71,072 records, 71,053 were not related (66125) and not as per the defined inclusion criteria (4,928). After the removal of non-relevant records, only 19 records were retrieved by the author for full text. No irretrievable article was found. After full-text screening of the 19 records, all 19 records were considered for the literature review, and two articles were considered for further situational analysis (Figure 1) [3].

Discussion

Historical Concept of Circular Building Through a Literature Review

The circular hospital design concept is not a new one; rather, it is a very old concept, dating back to 1878, as evidenced in literature by Snell (1886) [4]. The impactful thrust given to the entire world was by Professor John Marshall’s concept in the 1880s [5]. Thereafter, evidence for the circular hospital building concept was found in the writing of Jacobs and Williams and Srinivasan in the 1960s [6,7]. In 1988, Taylor critically reevaluated Jon Marshall’s concept of circular hospital building [5]. A recent study in 2015 by Steadman mentioned various circular plan buildings, including hospitals, and coined the term “doughnut” in the circular building concept [8].

In 1884, the first circular wards were established in England at the Miller Memorial Hospital in Greenwich, UK. The hospital included nine circular, one-story wards, each with 26 beds and a circumference of 66 feet. The circular ward concept was also used in London’s Great Northern Hospital; however, it lost its originality around 1890 [5].

**Figure 2:**
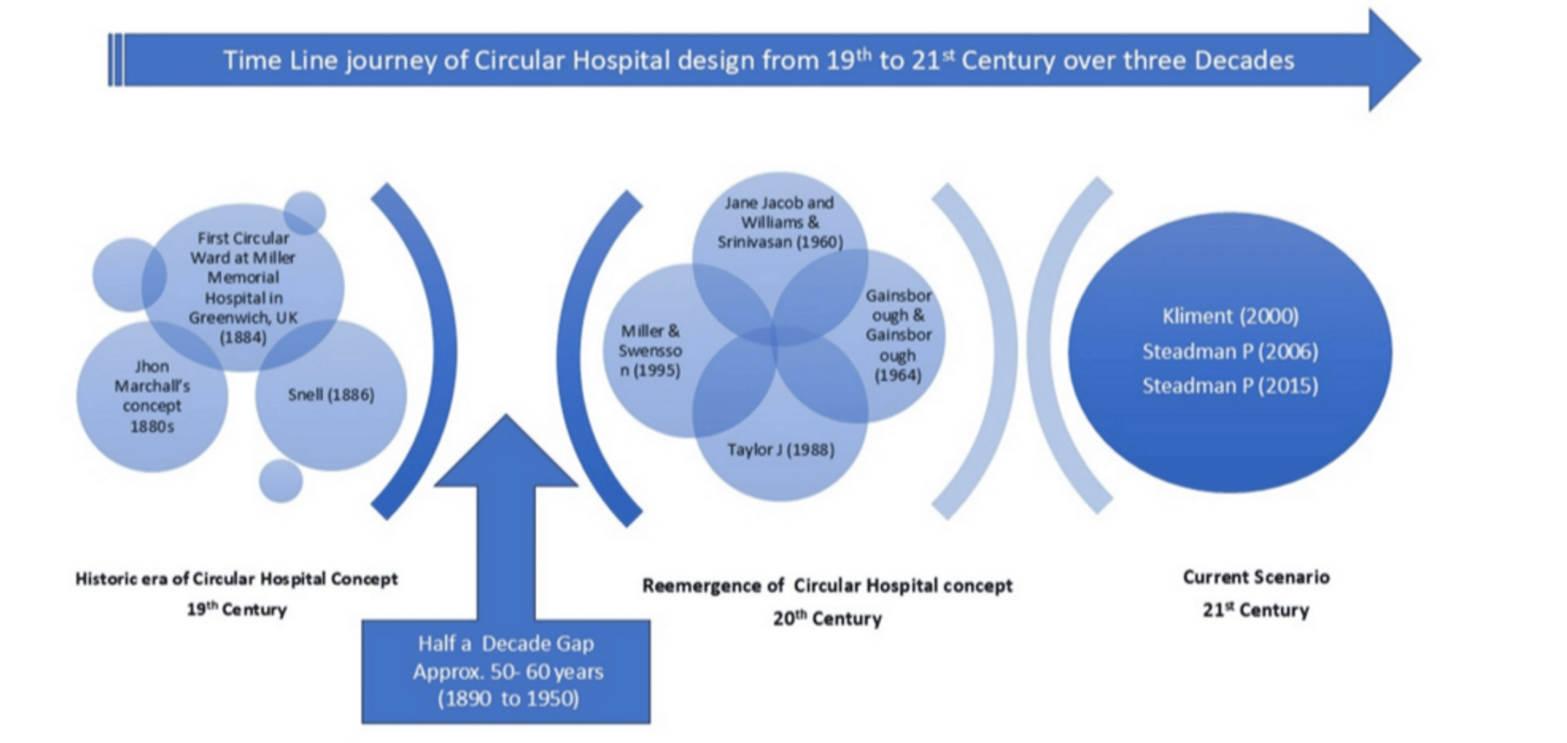
Timeline diagram related to circular hospital building design over three decades. The figure was created using MS PowerPoint software.

Several scholars, such as Gainsborough and Gainsborough (1964) [9], Miller and Swensson (1995) [10], Catananti et al. (1997) [11], Kliment (2000) [12], and many more [13-23] have contributed to nursing units design efficiency using a variety of factors such as flow patterns, compact shape, size, flexibility, adaptability to future changes, and construction and operating expense, as depicted in a illustrative timeline diagram (Figure 2). These are necessary for optimal operation, but further investigation is required [1].

Advantages mentioned in the literature include that nurses stationed at a central point can reduce travel time and energy and increase patient visibility. Circular units offer short travel distances and high visibility, making them more economical and efficient. Marshall presented the case for a circular ward plan, focusing on improved opportunities for light, air, and ventilation [13]. This geometry provides extra floor space and volume per bed, which can be compared to a rectangular format with the same number of beds and bed head spacing [5,7,8-23]. The disadvantages including circular construction is more expensive than rectangular construction in nursing units, but cost differences do not significantly affect design for efficiency and convenience. A large number of beds in a circular unit requires a large perimeter, increasing the central area. Snell was among those who opposed the circular ward plan for its vastness, wastefulness, and uncertainties regarding air circulation and sunlight [15,16]. Overall, the circular design has several merits such as visibility, efficiency, structural stability, and improved light and ventilation, except central core space wastage and construction complexity [5,6,8-23].

Which is Better? Circular, Elliptical, or Rectangular Building

The particular needs and objectives of the project determine the design of the building. Rectangular structures are favored for their simplicity of construction and space utilization, but circular buildings provide structural integrity, wind resistance, and energy efficiency. A balance between these two can be found in ellipsoidal forms, which give some benefits of each. Table 1 provides a summary of the elements for every shape [8-23].

**Table 1:** Comparison among rectangular, circular, and elliptical building properties.

Rectangular buildings	Elliptical buildings	Circular buildings
Pros: Simpler design and creation: Rectangular shapes are handy and economical as they are simple to plan and build. Flexible space use: Interior layouts are flexible and adaptable for applications, including commercial, industrial, and residential spaces, by linear and angular geometry. Easy to combine into existing urban fabric: Rectangular buildings maximize the use of available space on the common rectangular plots	Pros: Combination of benefits: Provides some of the aerodynamic benefits and visual appeal of circular buildings, but it may also allow for greater interior plan freedom than pure circles. Psychological impact: Curved curves are perfect for some buildings, such as homes and hospitals, because they create a feeling of intimacy and enclosure [24]	Pros: Enhanced stability: IRJET claims that a continuous, unbroken form is responsible for the uniform distribution of stress. Better seismic concert: Reduces torsional effects during seismic activity, ensuing in less shift and deformation than rectangular. Better wind resistance: By facilitating smoother airflow, the aerodynamic form lessens stress caused by wind. Aesthetically pleasing: Circular shapes frequently connected to grace, harmony, and a feeling of completion
Cons: stress concentration: Under seismic and wind loads, the angular design may cause stress concentrations at corners. Engineers have, nevertheless, created methods to lessen this. Less aerodynamic: Possibly more susceptible to wind-induced stress than curved geometries	Cons: More complex than rectangular: Compared to rectangular buildings, elliptical designs still require more complex design and construction methods. Material selection challenges: Careful material selection is necessary as not all materials can be bent without losing their structural integrity	Cons: Complex design and construction: Requires more complex calculations and specific methods to engineer curved walls and forms. Higher building costs: Complexity may result in a higher building time and resource requirement. Challenges with interior layout: Difficult to fit typical rectangular furniture and room configurations into a circular layout [25]
Advantages: Easier construction: As they are easier to frame, rectangular shapes may be less expensive and need less specialized labor. Efficient space use: In general, rectangular layouts make better use of internal space and are simpler to furnish. Familiarity: Rectangular structures are more prevalent and simpler as they representing foundation of most building standards and construction methods	Advantages: Compromise between circular and rectangular: Elliptical forms can blend the more recognizable spatial qualities of rectangular buildings with some of the structural advantages of circular ones. Good for specific applications: Buildings with particular aerodynamic needs or those that must reduce wind resistance may benefit from elliptical forms	Advantages: Superior structural strength: In general, circular designs can be more robust and provide superior resistance to lateral loads (wind and earthquakes). Improved aerodynamics: In addition to being more resilient to wind, circular buildings may use less energy for heating and cooling. Even stress distribution: By more equally distributing stress, circular geometries may lower the chance of structural failure
Disadvantages: Weaker structural integrity: Particularly in regions with strong winds, rectangular buildings may be more vulnerable to wind damage. Stress focuses: Rectangular buildings may become weaker as a result of concentrated stress zones created by corners	Disadvantages: Complex construction: Similar to circular buildings, elliptical constructions can be more costly and difficult to construct. Less effective space use: Even while ellipses are preferable to circles, they can nonetheless provide certain difficulties for furniture placement and interior design	Disadvantages: Complex creation: More sophisticated building methods and possibly higher expenses needed for circular constructions. Less efficient space use: For furniture and interior design styles, circular layouts are less effective. Less design flexibility: Circular designs may be less adaptable to various building layouts or site constraints

The purpose of the building, its aesthetics, time and cost limits, site and location constraints, and other considerations all influence the best possible building design [9,23,24]. In summary, the ideal shape varies depending on the particular situation. Rectangular buildings are a sensible and affordable option for the majority of common construction projects. However, circular or elliptical shapes might be more suitable if energy economy, wind resistance, or structural strength/stability are crucial. Particular design objectives, site circumstances, and preferred aesthetic will determine which is the “better” option between an elliptical and circular building [9-12,23-25].

The above discussion has shed light on the first research question, and it is evident that a circular hospital building is a better choice compared to rectangular and elliptical shapes, in many operational aspects of a hospital, except for the construction challenges.

Why are circular building plans rarely found in contemporary housing design?: Residential buildings with central plans have been built since prehistoric times, providing security and protection. Steadman studied rectangular shapes and created a typology for buildings with circular plans [13]. Szczegielniak compared circular plans to orthogonal plans, proving greater energy efficiency, lower construction costs, and maximum daylight use [26]. Szczegielniak [26] examined 24 contemporary single-family houses with circular plans, identifying seven basic types based on layout arrangement and living space organization. Circular residential buildings are less popular due to design, technology, and construction costs [8-16]. They cater to residents’ needs and are designed for specific locations. Research on circular buildings is limited due to limited access to graphic materials and a lack of architect involvement [26].

Which Type of Nursing Unit Design is Better Based on Size, Bed Number, etc.? Situational Analysis

To answer this second research question as further analysis, the author follows two studies by Kazanasmaz [1] and Steadman primarily [8]. In reference to Steadman [2,8] and Snell’s [4,15,16] general argument, the author here reexamines a different number of laid-out bedded nursing unit designs from eight to thirty-six. Accordingly, the author reexamines the above theoretical circular ward plan with additional modifications, including all other assumptions of the study, and compares three situations.

Situation 1: Simple, compact circular building hospital design: The modified assumptions are: (a) The building is preferably of a few floors, up to two, with or without an elevator and a ramp. (b) Each floor ward will have two equal halves with two separate nursing units. (c) Each half’s dimension will be distributed equally. (d) External radius is taken as similar to the Steadman study. (e) Each bed wall is 2.35 m, and the bed length is 2 m, similar to the Steadman study. (f) Each bed space is 4.7 m² compared to Steadman’s 4.4 m² Nightingale ward standard. (g) Two extra “bed spaces” are allowed within the bed space calculation for doors, similar to the Steadman study. Dimensional statistics for these alternatives are given in Table 2. The following formulas were used in Table 2:

$C = T - B$

$[C] = [T] - [B]$

where,

$[B] = 4.7 \times [n]$; $[T] = \frac{22}{7} \times [r]^2$

**Table 2:** Dimensional statistics for a simple compact circular building hospital.

Number of beds (n)	External radius (r) (m)	Bed area (B) (m²)	Bed area (B) % of total area (T)	Circulation area (C) (m²)	Circulation area (C) % of total area (T)
8 beds	3.8	37.6	83%	7.8	17%
10 beds	4.6	47.0	71%	19.5	29%
12 beds	5.4	56.4	62%	35.2	38%
14 beds	6.2	65.8	55%	53.1	45%
16 beds	6.9	75.2	50%	74.4	50%
18 beds	7.0	84.6	55%	69.4	45%
20 beds	7.8	94.0	49%	97.2	51%
22 beds	8.6	103.4	44%	129.0	56%
24 beds	9.9	112.8	37%	204.8	63%
26 beds	10.2	122.2	37%	204.8	63%
28 beds	11.0	131.6	35%	248.7	65%
30 beds	11.8	141.0	32%	296.6	68%
32 beds	13.0	150.4	28%	380.7	72%
34 beds	14.6	159.8	24%	510.1	76%
36 beds	16.2	169.2	21%	655.6	79%

**Figure 3:**
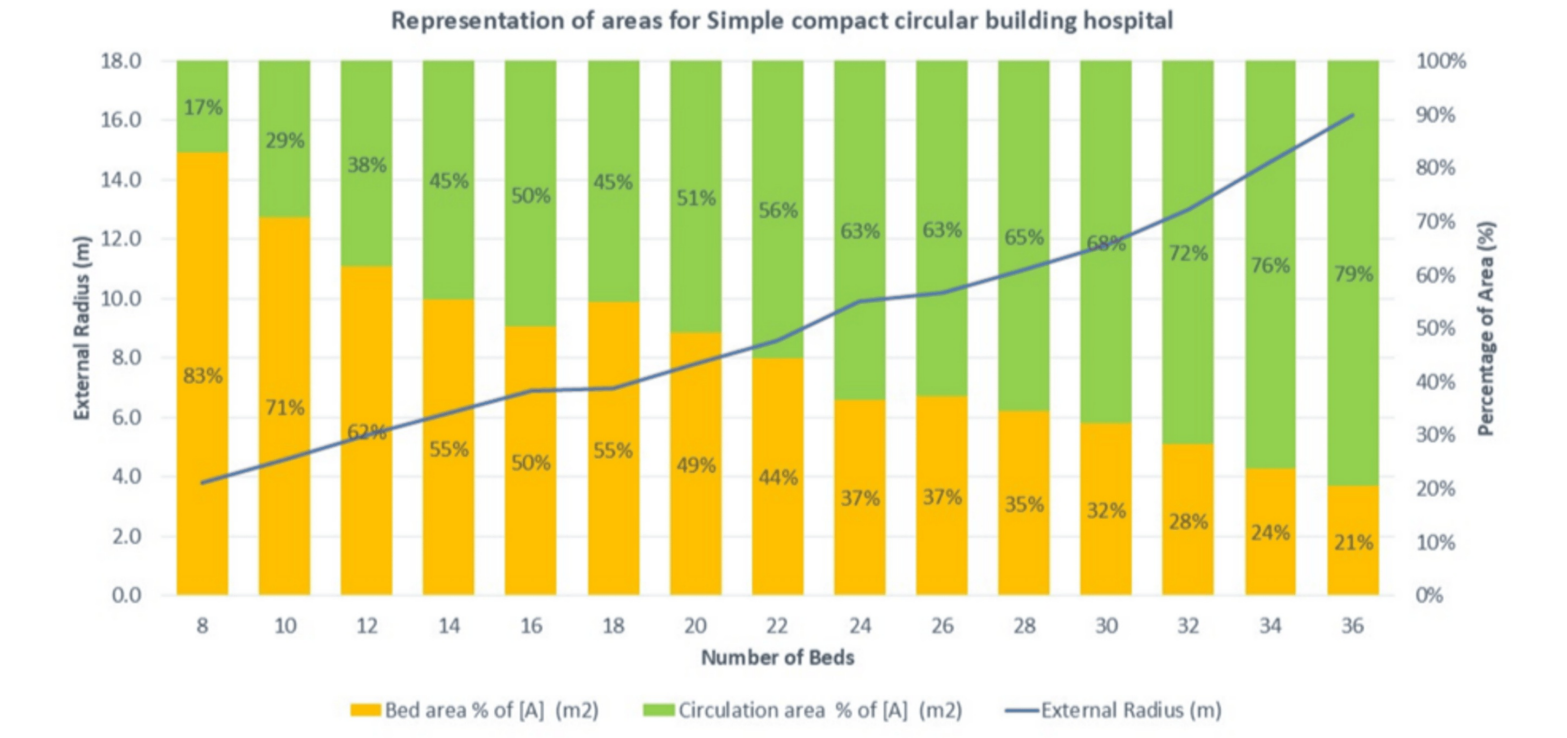
Representation of areas for a simple compact circular building hospital design. The figure was made using Microsoft Excel and PowerPoint software.

Here, similar to the study of Steadman [8], as the number of beds along with the external radius increases, circulation space increases and bed space decreases (Figure 3).

Snell, a former supporter, countered Marshall’s argument and argued that the capital and running costs of unused space in large circular wards were high [4,15,16]. Snell and other critics questioned the cost-effectiveness of eight- and twelve-bed designs, and the smaller plan; the feet of the beds are very close together [4,8,13,15,16,21]. For a larger plan, there were the issues of depth and difficulty in ventilation, beconing uncomfortable in summer conditions. Burdett also defended the design due to its function, ventilation, and use of available space [1,24,26]. The trend for circular wards lasted only two decades, with only a few small schemes built. Circular buildings, such as Marina City in Chicago and Washington, DC’s Cafritz office blocks [8]. These buildings, such as Capitol Records’ Los Angeles headquarters, have larger diameters, causing economic concerns for developers [8,13,15,16]. They also face planning issues, such as layout problems and higher construction costs due to specialized components and complex formwork [8,15,16]. Here, in this study, for this situation, a total of 20 to 26 beds, i.e., two nursing units with 10 to 13 beds each, is an economical and efficient design considering the best utilization of space.

**Figure 4:**
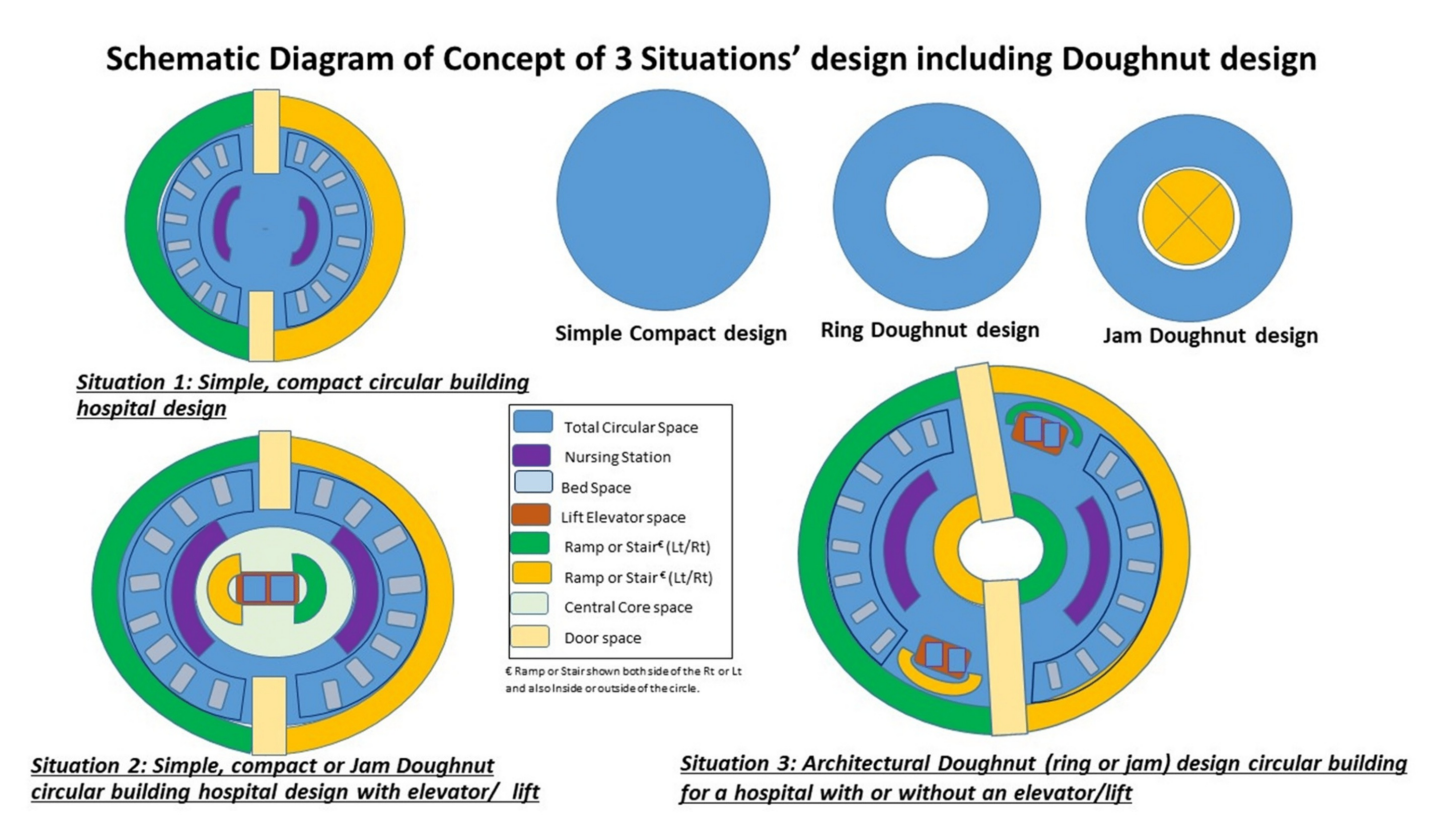
A schematic architectural diagram of three situations along with ring and jam doughnut concept. This schematic diagram was created using Microsoft PowerPoint software.

Architectural doughnuts are circular-shaped buildings with two types: ring doughnuts and jam doughnuts. A jam doughnut may be considered, but for structural construction safety and better aerodynamics, a ring doughnut is preferred [8]. Circular buildings were adopted by English hospital architects in the early 1880s for their simplicity and aesthetic potential [8,13,15,16]. The morphospace concept is used to study building plans and provides a percentage of total floor area represented by the outer ring. More efficient plans are found at the lower left, while less efficient ones are up and right. This tool allows architects to measure performance variations across theoretical space [8]. A schematic architectural diagram of three situations along with the doughnut concept is shown in Figure 4.

Situation 2: Simple, compact, or jam doughnut circular building hospital design with elevator or lift: The primary disadvantages of wastage of central core space is taken care by the author here with installation of elevator or lift at the central core space. After 1920, multistoried buildings began to appear. A study of a 14-storey circular building with central toilet and lifts was proposed for a hospital by Williams and Srinivasan [7], which minimizes wasted space, reduces nurses’ walking, maximizes space, offers ventilation and roof protection, and increases planning efficiency. Vertical transportation is cheaper in two- or three-storey buildings with a ramp compared to an elevator, as described by Srinivasan in child hospital design in South India [5,7].

The modified assumptions, in addition to Situation 1, are: (a) The calculations were made for each floor only. (b) Multiple floors of maximum seven with fire safety compliance and without a ramp. (c) Minimum two elevators, considering patient safety and efficiency. (d) Space for each elevator is 15.5 m² as per a commercial building. (e) Minimum dimension of each bed space is 4.7 m². (f) Each elevator accommodates two beds and accompanying person and equipment. (g) Any calculated negative value area is considered as 0%. (h) The elevator will be placed at the inner core of the circle. Different dimensional statistics are provided in Table 3. The following formulas were used in Table 3:

$[C']=[T]-[B]-[E]$

where,

$[B] = 4.7 \times [n], \qquad[T] = \frac{22}{7} \times [r]^2, \qquad[E] = [e] \times 15.5$

**Table 3:** Dimensional statistics for a simple compact circular building hospital design with an elevator.

Number of beds (n)	External radius (r) (m)	Bed area (B) % of total area (T)	Number of elevators/lifts proposed (e)	Elevator/lift area (E) (m²)	Elevators/lifts area (E) % of total area (T)	Proposed circulation area (C') (m²)	Adjusted circulation area (AC) % of total area (T)
8 beds	3.8	83%	2	31.0	68%	-23.2	0%
10 beds	4.6	71%	2	31.0	47%	-11.5	0%
12 beds	5.4	62%	2	31.0	34%	4.2	5%
14 beds	6.2	55%	3	46.5	39%	6.5	6%
16 beds	6.9	50%	3	46.5	31%	27.9	19%
18 beds	7.0	55%	3	46.5	30%	22.9	15%
20 beds	7.8	49%	4	62.0	32%	35.2	18%
22 beds	8.6	44%	4	62.0	27%	67.0	29%
24 beds	9.9	37%	4	62.0	20%	133.2	43%
26 beds	10.2	37%	5	77.5	24%	127.3	39%
28 beds	11.0	35%	5	77.5	20%	171.2	45%
30 beds	11.8	32%	5	77.5	18%	219.1	50%
32 beds	13.0	28%	5	93.0	18%	287.7	54%
34 beds	14.6	24%	7	108.5	16%	4.1.6	60%
36 beds	16.2	21%	8	124.0	15%	531.6	64%

**Figure 5:**
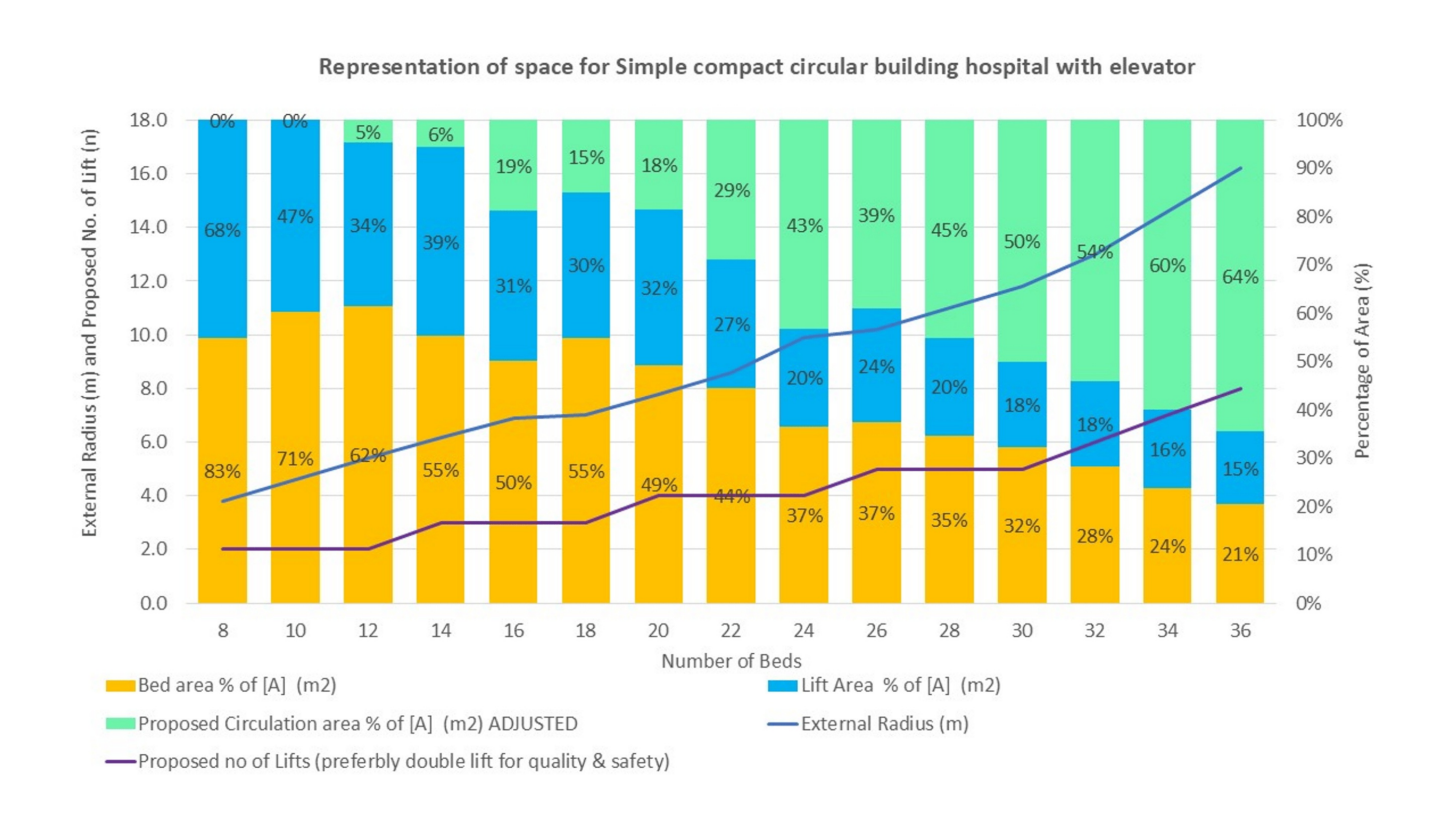
Representation of space for a simple compact circular building hospital with an elevator. The figure was created using Microsoft Excel and PowerPoint software.

Here, one can find that the space pattern is similar to the previous situation, with increasing bed or radius circulation area (Figure 5). Smaller circular plans are not feasible or have very little circulation space, specifically for 8-16-bedded spaces. They will not be economically viable either.

Further, the larger the circular plan, specifically beyond 34 beds, the more there is wastage of space. With respect to better space utilization with elevator space at the central inner core of the circular building, the bed range of 24 to 30, i.e., two nursing units each with 12 to 15 beds, may be considered. However, in high-rise multistorey buildings, other structural issues such as fire safety, footfall, and provision of ramps need to be considered.

Situation 3: Architectural doughnut designs a building for a hospital with or without an elevator/lift: In addition to the assumptions of Situations 1 and 2, the maximum possible inner ring radius was considered by deducting the two-bed length from outer radius, i.e., 2 m only, e.g., for a smaller eight-bedded circular plan, the maximum radius for the inner core is 1.8 m only (Table 4) was added. In this situation, two variants with or without an elevator/lift were considered. This situation was considered to show the ring doughnut design specifically; however, the jam doughnut design is also feasible (Figure 4). The following formulas were used in Table 4:

$[C'&lsquo;] =[T]-[B]-[R]$

And

$[AC&rsquo;] = [C&rdquo;]-[E]$

Where, $[AC'] = [C''] - [E],\qquad[B] = 4.7 \times [n],\qquad[T] = \frac{22}{7} \times [r]^2,\qquad[E] = [e] \times 15.5,\qquad[R] =\frac{22}{7} \times [r']^2$

**Table 4:** Dimensional statistics for the Doughnut design building hospital with or without an elevator.

Number of beds (n)	External radius ® (m)	Bed area (B) % of total area (T)	Number of elevators (e)	Elevator/lift area € (m²) % of total area (T)	Inner core radius (r’) (m)	Inner core area (R) (m²) % of total area (T)	Proposed circulation area (C’’) (m²) % of total area (T)	Adjusted circulation area (AC’) % of total area (T)
8 beds	3.8	83%	2	68%	1.8	12%	25%	0%
10 beds	4.6	71%	2	47%	2.6	12%	17%	0%
12 beds	5.4	62%	2	34%	3.4	12%	27%	0%
14 beds	6.2	55%	3	39%	4.2	11%	34%	0%
16 beds	6.9	50%	3	31%	4.9	10%	39%	8%
18 beds	7.0	55%	3	30%	5.0	10%	35%	5%
20 beds	7.8	49%	4	32%	5.8	10%	41%	9%
22 beds	8.6	44%	4	27%	6.6	9%	47%	20%
24 beds	9.9	37%	4	20%	7.9	8%	55%	35%
26 beds	10.2	37%	5	24%	8.2	8%	55%	31%
28 beds	11.0	35%	5	20%	9.0	7%	58%	38%
30 beds	11.8	32%	5	18%	9.8	7%	61%	43%
32 beds	13.0	28%	6	18%	11.0	7%	65%	48%
34 beds	14.6	24%	7	16%	12.6	6%	70%	54%
36 beds	16.2	21%	8	15%	14.2	5%	74%	59%

**Figure 6:**
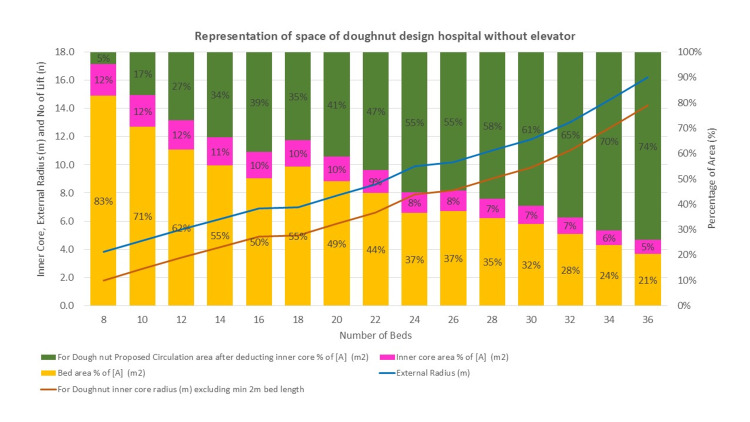
Representation of the space of a doughnut design hospital without an elevator. The figure is created by Microsoft Excel and PowerPoint software.

**Figure 7:**
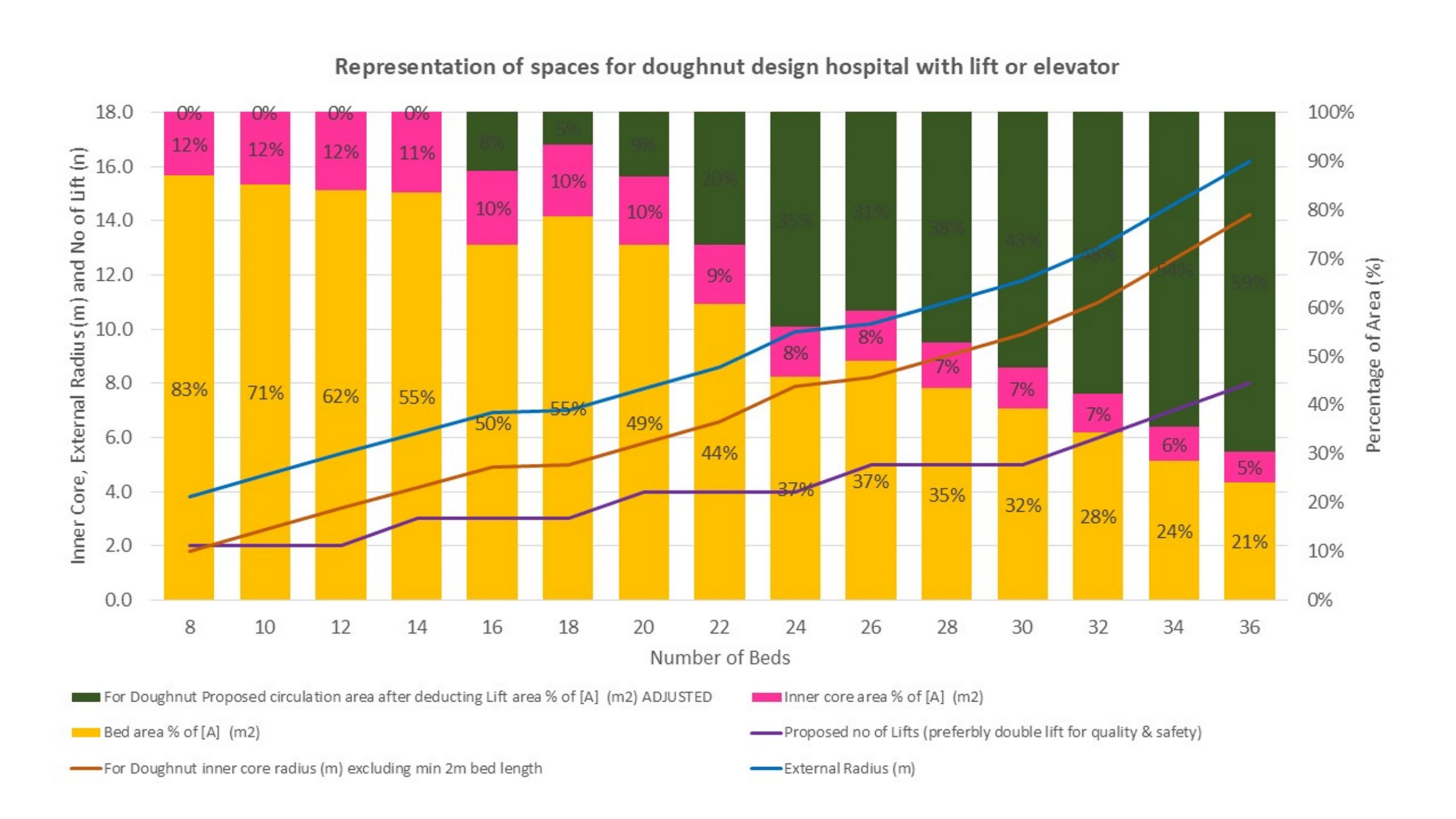
Representation of spaces for a doughnut design hospital with a lift or elevator. This figure is created using Microsoft Excel and PowerPoint software.

The space distribution for the doughnut design with or without a lift or elevator is shown in Figure 6 and Figure 7.

In both cases, it is visible that better space utilization is possible with the increasing bed number, and considering the centralized inner core lift/elevator, the bed number range further increases. The circulation area can increase further, depending on the inner core diameter considered. Examples of such buildings are Apple’s headquarters and Rajkumari Ratnavati School in Rajasthan, India. Larger the plan of doughnut design, the more structurally, aerodynamically stable, economically viable, and eco-friendly it is. Though a ramp is not considered here, but it is evident that with larger circular building, a C-shaped ramp can be possible with both inner circle as well as outer circle areas (Figure 4).

In terms of crowd management and better space utilization, considering bed: circulation area = 1:1.5; the minimum possible bed numbers for circular, circular with elevator, doughnut, and doughnut with elevator are 24, 30, 24-26, and 32, respectively, divided equally in two nursing units on each floor.

Study limitations

The study is primarily based on a literature review of PubMed, Scopus, Web of Science, and Google Scholar databases only; grey literature was excluded. The extended study of Steadman was based on several assumptions. Only the elevator factor was considered here; however, many more factors are related to the hospital design or nursing unit design, which require further investigation.

Future directions

With the advancement of modern architectural engineering, construction of any building becomes easier. Here, the author has presented a concept of a circular hospital building with details of its pros and cons. However, further investigation is required in different aspects of load capacity, visibility, ventilation, strength, stability, patient safety, aerodynamic effect, and thermodynamic effect, using different engineering software. The author would like to develop an AutoCAD model of the suggested designs and simulate different parameters in simulation software (e.g., Ansys), specifically for aerodynamic and thermodynamic studies in the future. Overall, the circular design may matter in modern healthcare systems, and it could also be strengthened with contemporary examples related to infection prevention, workflow optimization, sustainability, or patient-centered design.

## Conclusions

A better hospital design depends on many factors. In this review, the author concluded that circular building design has tremendous potential in hospital or nursing unit design owing to its properties of strength, stability, air dynamics, visibility, ventilation, and patient safety compared to elliptical and rectangular shapes. For a compact circular hospital building, a plan that is too small or too large, with or without an elevator, will not be structurally and economically viable. Whereas the doughnut design will be economically and structurally more viable for a larger circular plan. With the introduction of a central core elevator/lift, not only is the wastage of central space reduced, but the bed capacity will also increase for both circular and doughnut designs. The smaller compact circular design may be very useful and planned for places where land cost is high and not easily available, and the larger doughnut design can be useful where land cost is cheap and easily available. Modern construction engineering with advanced technologies such as pneumatic tube, capsule transportation system, centralized HVAC system, MGPS system, and other modern amenities not only makes the construction simple but adds value and reduces cost in the long term. Delay due to transportation can be avoided, leading to timely supply chain management and reducing travel time, manpower requirement, and chance of human error, adding value to patient safety and quality service in healthcare. This is the design choice for the future. However, this circular hospital or nursing unit design requires further in-depth research in this unexplored field with a multidisciplinary approach.
